# Temperature Sensitivity of Wild-Type, Mutant and Genetic Sexing Strains of *Ceratitis capitata*

**DOI:** 10.3390/insects13100943

**Published:** 2022-10-18

**Authors:** Germano Sollazzo, Georgia Gouvi, Katerina Nikolouli, Elena I. Cancio Martinez, Marc F. Schetelig, Kostas Bourtzis

**Affiliations:** 1 Insect Pest Control Laboratory, Joint FAO/IAEA Centre of Nuclear Techniques in Food and Agriculture, Department of Nuclear Sciences and Applications, IAEA Laboratories, 2444 Seibersdorf, Austria; 2 Department of Insect Biotechnology in Plant Protection, Institute for Insect Biotechnology, Justus-Liebig-University Gießen, Winchesterstr. 2, 35394 Gießen, Germany; 3 Department of Environmental Engineering, University of Patras, 2 Seferi Str., 30100 Agrinio, Greece

**Keywords:** Mediterranean fruit fly, sterile insect technique, temperature-sensitive lethal, white pupae, Tephritidae, Diptera

## Abstract

**Simple Summary:**

The Mediterranean fruit fly (medfly), *Ceratitis capitata*, is a major agricultural insect pest species worldwide. The sterile insect technique (SIT), as a component of area-wide integrated management (AW-IPM) programmes, is currently used to control populations of this pest. SIT is based on the mass production and release of sexually sterile insects, ideally males, over a target area. Male-only releases can be achieved by using genetic sexing strains (GSS) such as the medfly VIENNA 8 GSS. Females of this strain are homozygous for a mutation in the *temperature-sensitive lethal* (*tsl*) gene, which kills them when exposed to high temperatures during the embryonic stage. In the present study, we employed a temperature-sensitive lethal test (TSLT) to determine the temperature sensitivity or tolerance of twenty-seven *Ceratitis capitata* wild-type, genetic sexing, and *tsl* mutant strains. Significant differences were detected among the strains studied with respect to egg hatching, pupal, and adult recovery rates. We discussed our findings in the context of SIT applications and climate change.

**Abstract:**

Area-wide integrated pest management (AW-IPM) programmes with a sterile insect technique component (SIT) are used to control populations of insect pests worldwide, including the Mediterranean fruit fly, *Ceratitis capitata*. SIT consists of the mass rearing, radiation-induced sterilization, handling, and release of sterile insects over the target area. Although SIT can be performed by using both sterile males and females, male-only releases significantly increase the efficiency and cost-effectiveness of SIT applications. Male-only releases can be achieved by using genetic sexing strains (GSS). The medfly VIENNA 8 GSS is based on two selectable markers, the white pupae (*wp*) gene, and the temperature-sensitive lethal (*tsl*) genes. The latter allows the elimination of females by exposing embryos to elevated temperatures. This study assessed the temperature sensitivity of twenty-seven medfly strains through a TSLT. Our results indicated significant differences among the strains regarding egg hatching as well as pupal and adult recovery rates due to the presence or absence of the *tsl* mutation and/or the genetic background of the strains. Our findings are discussed in the context of SIT applications, the importance of the *tsl* gene for developing genetic sexing strains, and climate change.

## 1. Introduction

The Mediterranean fruit fly, *Ceratitis capitata* (Wiedemann, 1824), also called “medfly”, is a member of the Tephritidae family in the order Diptera and is considered one of the most harmful species for agriculture worldwide. It is a cosmopolitan, highly polyphagous, and carpophagous species, and its presence has been documented in more than 260 species of fruits and vegetables of major economic interest [1,2]. Medfly’s rapid invasion in five different continents and its extensive destructive potential have fueled intensive control programmes aiming to restrict the distribution of the pest in the invaded areas [3,4]. Several methods have been developed to control medfly populations including the sterile insect technique (SIT) [5,6,7].

As a component of area-wide integrated pest management programmes (AW-IPM), the sterile insect technique has been used to control populations of insect pests and disease vectors worldwide [8]. SIT is based on the mass production and release of irradiation-induced sterile flies over a target area to suppress wild populations of a target species [5,7]. During the last decades, AW-IPM programmes with an SIT component have been developed and implemented to control populations of insect pest species, including medfly, in several parts of the world with the purpose of suppression, prevention, containment, or eradication strategies [9,10,11,12,13].

Several studies have shown that the mass production and release of sterile males only, increase the efficiency and cost-effectiveness of SIT applications significantly. This is because: (a) costs associated with the mass production, handling, release, and post-release monitoring activities are reduced, as there is no production, handling, and release of females, (b) released sterile males are not engaged in mating with sterile females and are only seeking wild females, and (c) avoiding releases of sterile females prevents further damage of fruits and vegetables [14,15,16,17]. An efficient and robust way to separate males from females is through developing and using genetic sexing strains (GSS) [17].

**Figure 1:**
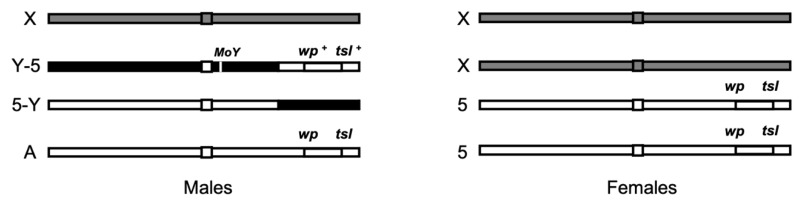
Schematic representation of a *Ceratitis capitata* VIENNA GSS. GSS females are homozygous for the mutant alleles of the *wp* and *tsl* gene markers, while GSS males are heterozygous at both loci with the wild-type alleles of the two markers linked to the male determining region of the Y chromosome (MoY) through an irradiation-induced T(Y;5) translocation.

Two GSS are currently used in mass rearing facilities to produce sterile males for SIT applications against medfly, namely VIENNA 7 and VIENNA 8 [18]. Both of them have been constructed using classical genetic approaches and carry two recessive selectable markers, the *white pupae* (*wp*) and the *temperature-sensitive lethal* (*tsl*) genes, which are localized on the right arm of chromosome 5 [17]. The females of the VIENNA 7 and VIENNA 8 GSS are homozygous for the mutant alleles of the *wp* and *tsl* genes, emerge from white puparia, and die when exposed as 24-h-old embryos to elevated temperatures (34–35 °C) for 24 h. The GSS males are heterozygous on both genetic loci, with wild-type alleles linked to the Y chromosome and the male determining region through an irradiation-induced translocation. They emerge from brown puparia and survive when exposed to high temperatures [17,19] (Figure 1). The only difference between VIENNA 7 and VIENNA 8 GSS lies in the translocation. VIENNA 7 and VIENNA 8 GSS carry the translocation at position T(Y;5)52A and T(Y;5)58B, respectively (translocation breakpoints determined based on trichogen polytene chromosome maps) [17,19]. Due to chromosomal translocation, GSS are considered semi-sterile presenting reduced productivity, which depends on how the Y-autosome translocation segregates during gametogenesis. Alternate segregation produces genetically balanced progeny, while adjacent-1 segregation produces unbalanced progeny due to deletions and triplications. Progeny with deletions usually die at the embryonic stage. However, those with triplications may survive even up to the pupal or the adult stage, which may explain the difference in the productivity levels observed between different GSS [17].

Recombination phenomena may affect the genetic stability of a GSS and, through the accumulation of recombinants during mass-rearing, can result in the loss of their genetic sexing properties [17,19]. In medfly, this problem has been addressed by: (a) the development and implementation of a “filter rearing system”, which removes the recombinants and thus maintains the genetic integrity of the GSS [15,20] and (b) the development of the In(5)50C;59C inversion on chromosome 5 (inversion breakpoints determined based on trichogen polytene chromosome maps), which is also known as “D53 inversion”), that carries the *wp* and *tsl* selectable markers. Inversions are known suppressors of genetic recombination and the incorporation of the D53 inversion in the VIENNA GSS has significantly increased their genetic stability [17,19].

A recent study assessed the genetic stability and rearing efficiency of VIENNA 7 and VIENNA 8 GSS at different facilities worldwide [18]. Despite their common origin, the strains showed differences in egg hatching, pupal recovery, adult emergence rates, and male tolerance to elevated temperatures [18]. The causal factor(s) for these differences could be environmental and/or genetic. The productivity of the GSS may vary in laboratories and mass-rearing facilities due to different rearing practices, including the type of diet and type of translocation and its segregation behaviour during gametogenesis [17,18]. Long-term colony maintenance and inbreeding and the refreshment of mass-rearing colonies with wild genetic material followed by adaptation in the given rearing conditions may have resulted in altered phenotypic properties, with respect to sex separation and male recovery rates upon heat treatment, both being critical for the mass production of high-quality males for SIT applications [17,18]. Thus, in this study, we assess the temperature sensitivity and recovery rates of several wild-type, mutant, and GSS medfly strains under small-scale, laboratory rearing conditions. 

## 2. Materials and Methods

### 2.1. Ceratitis Capitata Strains and Rearing Conditions

All strains were maintained under small-scale, laboratory rearing conditions at 24 ± 2 °C, 55 ± 10% RH, and 14/10 h light/dark cycle at IAEA Insect Pest Control laboratories (Seibersdorf, Austria). Adults were placed in 20 × 20 × 30 cm cages and fed on yeast: sugar (1:3) with water being provided separately. Larvae were reared on a carrot diet as described previously [21] with minor modifications: 2.5 kg carrot powder (Van Drunen Farms, Momence, IL., USA), 7 kg cooked frozen baby carrots (Ardo, Ardooie, Belgium), 840 g yeast hydrolysate enzymatic (MP Biomedicals^TM^), 80 g sodium benzoate, water up to 10 L while the pH was adjusted to 4.3–4.4 with 115 ml of hydrochloric acid 32%. As shown in Table 1, twenty-seven strains were used in the present study, classified into three groups: “wild type” strains (No. 1–9), “GSS” (No. 10–20), with females being homozygous and males being heterozygous for the *tsl* mutant allele, respectively, and “mutant” strains (No. 21–27) with all individuals being homozygous for the *tsl* mutant allele. The GSS strains were constructed by crossing males from the translocation lines (VIENNA 7, VIENNA 8) with females from the *wp tsl* mutant strain. VIENNA 7 and VIENNA 8 GSS have been reconstructed in different years and/or places; this is indicated in their names. Additional characteristics for some of these strains are presented in Table 1 as footnotes. 

### 2.2. Temperature-Sensitive Lethal Test 

**Figure 2:**
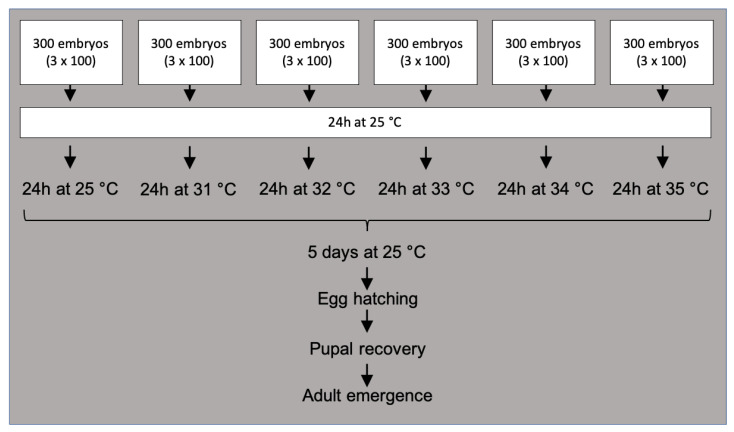
TSLT workflow. For each temperature test, 300 freshly collected eggs were used and incubated at 25 °C for 24 h. After that, heat treatments were conducted for 24 h, and the egg hatching, pupal recovery, and adult emergence rates were determined.

The TSLT was performed as previously described [18] with the only difference that the GSS and the mutant strains were tested in all six temperatures (25, 31, 32, 33, 34, and 35 °C) while the wild-type strains were only assessed at 25, 34 and 35 °C (“short TSLT”). Each TSLT was performed with 100 eggs per temperature and replicated three times by collecting eggs over three consecutive days. In total, 1800 eggs (100 eggs × 3 replicates × 6 temperatures) were used for the “TSLT”, while 900 eggs were used for the “short TSLT”. All eggs were collected within five hours from adult cages containing 5 to 7 days old adults. After collection, eggs were placed on black strips (100 eggs/strip) on top of 90 × 15 mm Petri dishes with larval carrot diet and held at 25 °C for 24 h. After 24 h incubation, each batch was kept at one of the six different temperatures (25, 31, 32, 33, 34, and 35 °C). After the heat treatment, all eggs were left at 25 °C to complete their development. Egg hatching, pupal recovery, and adult emergence rates were determined 5, 15, and 23 days post egg collection, respectively (Figure 2). Egg hatching was calculated for each replicate using the number of collected embryos (100) as a reference and the number of empty eggshells after five days. Pupal recovery was calculated using the number of pupae obtained divided by the hatched eggs. The adult recovery rate was calculated using the number of eclosed adults divided by the number of pupae.

### 2.3. Statistical Analysis

All statistical analyses were performed using R version 4.0.5 (R Core Team 2021). Egg hatching, pupal recovery, and adult emergence rates represent recovery ratios. Therefore, all datasets were analyzed using a GLM-binomial family and a logit link function [23]. For the recovery rates, the following parameters were measured: a) egg hatching, b) pupae recovery, and c) adult recovery, as the number of adults emerged from the total number of pupae. The generalized linear models (GLM) overdispersion was checked with the DHARMA package [24]. DHARMA tests if the simulated dispersion is equal to the observed dispersion and supports the visual inspection of the residuals. Overdispersion of the GLM-binomial models was addressed with a quasi-binomial model using a logit link function [25]. Analysis of deviance was performed with a Chi-squared test for GLM-Binomial models and an F-test for GLM-quasi-binomial models [26]. Half-normal plots with simulation envelopes were used to inspect the goodness-of-fit of the models [27] visually. The pairwise comparisons of the fitted model estimates were calculated with the ‘estimated marginal means’ (emmeans) package [28]. For all data, the significance level was set to α = 0.05.

## 3. Results

### 3.1. Egg Hatching at 25 °C, 34 °C, and 35 °C

**Figure 3:**
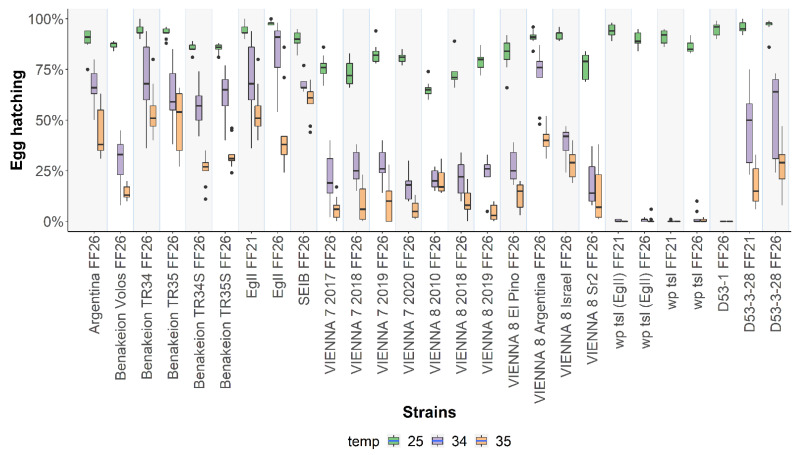
Egg hatching rates of twenty-seven *Ceratitis capitata* wild-type, mutant, and GSS strains. Egg hatching rates of strains reared at 25 °C without heat-shock treatment and after 24 h heat-shock treatment at 34 °C and 35 °C are shown. Dots represent outliers, while dashes represent the absence of variance in the data.

Statistically significant differences were detected among the strains for egg hatching at the rearing temperature of 25 °C (F = 24.851, *df* = 26, *p* < 2.2 x 10^-16^) (Figure 3; Table S1). Regarding the wild-type strains, egg hatching ranged from 85.11 ± 2.76% for Benakeion TR 34S to 98 ± 1% for EgII FF26 at 25 °C. In the case of GSS, egg hatching at 25 °C had its lowest value at 65.22 ± 4.12% for VIENNA 8 2010 and reached up to 92 ± 2.40% for VIENNA 8 Israel. The respective values for the mutant strains were 86.22 ± 2.91% for wp tsl FF26 and 96.11 ± 4.04 for D53-3-28 FF26. Significant differences were also observed among all strains when embryos were exposed for 24 h at both 34 °C (F = 48.767, *df* = 26, *p* < 2.2 x 10^-16^) and 35 °C (F = 40.615, *df* = 26, *p* < 2.2 x 10^-16^) (Figure 3). Heat exposure at 34 °C had moderate effects on egg hatching for the wild-type strains and more pronounced ones in the case of the GSS and mutant strains. Mutant strains had a hatching rate of zero or close to zero at 34 or 35 °C, which manifests the thermosensitivity of the *tsl* mutant allele present in these strains, except for the D53-3-28 FF21 and D53-3-28 FF26 strains. Similarly, recovery of females was reduced in the GSS as they were homozygous for the *tsl* mutant allele (Table S1). Pairwise comparisons of each strain are shown in Table S2.

### 3.2. Pupal Recovery at 25 °C, 34 °C, and 35 °C

**Figure 4:**
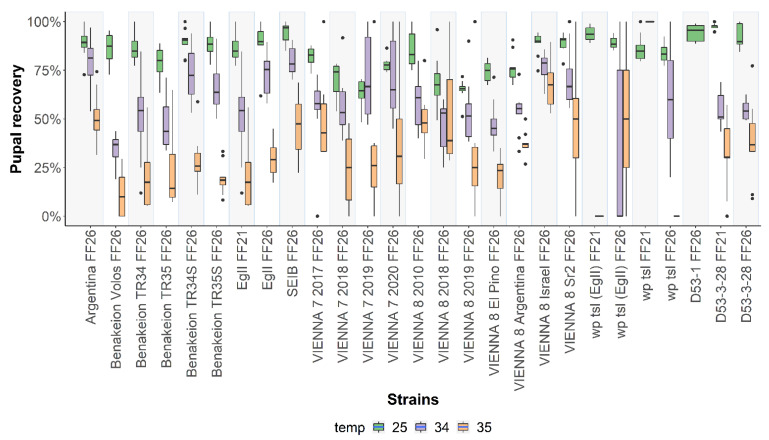
Larval to pupal recovery rates of twenty-seven *Ceratitis capitata* wild-type, mutant, and GSS strains. Pupal recovery rates of strains reared at 25 °C without heat-shock treatment and after 24 h heat-shock treatment at 34 °C and 35 °C are shown. Dots represent outliers, while dashes represent the absence of variance in the data. In the case of *wp tsl* (EgII) FF21, *wp tsl* FF21, and D53-1 FF26, the missing data are due to undefined ratios (denominator was 0).

Pupal recovery rates were assessed based on the hatched larvae for all strains evaluated. Statistically significant differences were detected among all strains tested in respect to pupal recovery at 25 °C (F = 12.944, *df* = 25, *p* < 2.2 x 10^-16^) and ranged between 50.11 ± 9.1% for VΙΕNNA 8 2018 and 93.22 ± 3.53% for D53-3-28 FF21 (Figure 4; Table S1). The low pupal recovery of the GSSs was attributed to their semi-sterility. Significant differences were also observed when embryos were exposed for 24 h at both 34 °C (F = 6.241, *df* = 24, *p* = 5.011 x 10^-14^) and 35 °C (F = 5.5303, *df* = 22, *p* = 2.266 x 10^-11^) (Figure 4; Table S1). The highest pupal recovery rates among wild-type strains were observed in EgII FF26 (59.78 ± 13.54%), while the lowest was observed in Benakeion Volos (10.56 ± 5.41%), when embryos were exposed at 34 °C. VIENNA 8 Argentina and VIENNA 8 2018 had the highest and lowest pupal recovery at 34 °C among the GSSs, respectively, while in the case of the *tsl* mutant strains, heat-shock at 34 °C had detrimental effects in all strains with 0 or close to 0 pupal recovery (Table S1). As mentioned above, D53-3-28 FF21 and D53-3-28 FF26 were the only *tsl* mutant strains that exhibited an abnormal pattern and behaved unlike their mutant classification. Pairwise comparisons of each strain are shown in Table S3, indicating statistically significant differences among the wild-type strains, GSS, and mutant strains.

### 3.3. Adult Recovery at 25 °C, 34 °C, and 35 °C

**Figure 5:**
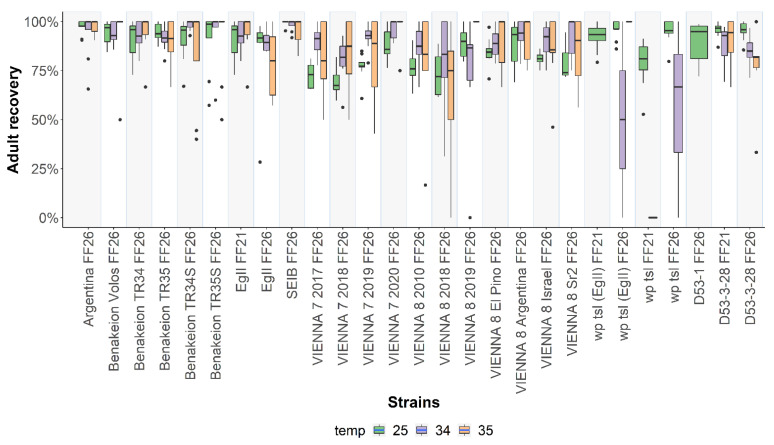
Pupal to adult recovery rates of twenty-seven *Ceratitis capitata* wild-type, mutant, and GSS strains. Adult recovery rates of strains reared at 25 °C without heat-shock treatment and after 24 h heat-shock treatment at 34 °C and 35 °C are shown. Dots represent outliers, while dashes represent the absence of variance in the data. In the case of *wp tsl* (EgII) FF21, *wp tsl* FF21, *wp tsl* FF26, and D53-1 FF26, the missing data are due to undefined ratios (denominator was 0).

Similar to what was observed for egg hatching and pupal recovery, statistically significant differences were also found among the strains studied with respect to adult recovery at 25 °C (F = 6.937, *df* = 25, *p* < 2.2 x 10^-16^; Figure 5; Table S1). Significant differences were also detected in the adult emergence when embryos were exposed for 24 h to both 34 °C (F = 2.5586, *df* = 26, *p* = 0.0002887) and 35 °C (F = 2.5149, *df* = 21, *p* = 0.0006646) (Figure 5; Table S1). Pairwise comparisons of each strain are shown in Table S4.

### 3.4. Temperature-Sensitive Lethal Tests at Additional Temperatures

**Figure 6:**
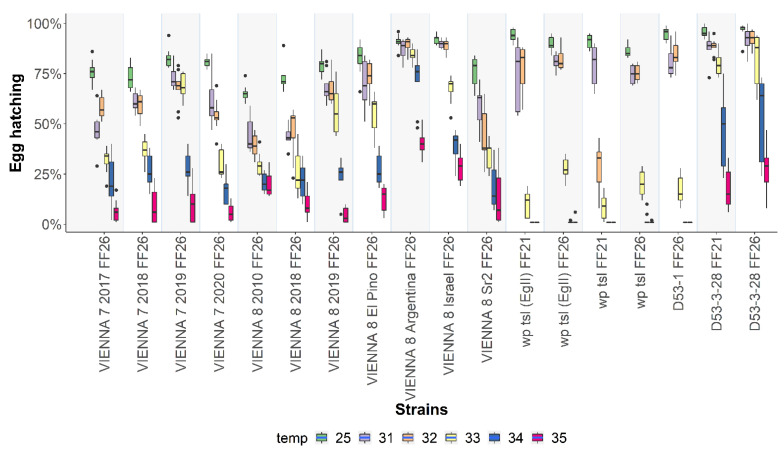
Egg hatching rates of *Ceratitis capitata* GSS and mutant strains of the TSLT at six different temperatures are shown. Dots represent outliers, while dashes represent the absence of variance in the data.

Heat exposure of 24 h old GSS and *tsl* mutant embryos at 34 °C and 35 °C showed differential sensitivity among the strains (Table S1). Due to this variability, we investigated the thermal response of these strains (GSS and mutant) at three additional temperatures, 31 °C, 32 °C, and 33 °C, to define the point at which heat sensitivity is expressed in each strain. Interestingly, egg hatching between 31 °C and 32 °C did not show any significant decrease, except for the strains VIENNA 7 2017 (df = 5, *p* = 0.0137), VIENNA 8 Sr^2^ (df = 5, *p* = 0.0377), and wp *tsl* FF21 (df = 5, *p* = < 0.0001), where the difference was statistically significant. When the temperature was increased to 33 °C, embryos presented higher sensitivity in most cases, apart from the strains VIENNA 7 2019, VIENNA 8 Sr^2^, and VIENNA 8 Argentina, which showed no significant decrease in egg hatching. The results indicated that the reduced fertility at the embryonic stage starts at 31 °C for all the GSS and *tsl* mutant strains except for VIENNA 8 Israel, VIENNA 8 Argentina, and D53-3-28 FF26, where exposure to 33 °C is required (Figure 6, Table S5). 

## 4. Discussion

The *temperature-sensitive lethal (tsl)* gene plays a crucial role in the successful implementation of SIT applications against the agricultural pest, *Ceratitis capitata*, as its engineered presence in the genetic sexing strains VIENNA 7 and VIENNA 8 allows the robust and efficient sex separation at the embryonic stage, thus resulting in the release of sterile males-only individuals [17,26,28]. As the function of the *tsl* gene is critical as a selectable marker for sex separation, its genetic stability should be monitored regularly to ensure the overall stability and robustness of the GSS [18]. The present study applied to different wild-type, GSS and *tsl* mutant strains temperature-sensitive lethal tests and confirmed, with some exceptions discussed below, the presence and stability of the *tsl* gene marker in several genetic sexing strains and *tsl* mutant strains that are routinely used in SIT-related research and applications. 

Temperature-sensitive lethal tests were performed on eleven GSS. Our results indicated that the thermal exposure of embryos reduced egg hatching, pupal recovery, and adult emergence. As female embryos were homozygous for the *tsl* allele and sensitive to elevated temperatures, lethality was observed mainly in females. In contrast, lethality was rescued in male embryos carrying a wild-type, *tsl^+^* allele. Moreover, a frequent application of “TSLTs”, in addition to the “filter rearing system” is strongly recommended due to the possibility of having recombinants, which, being white puparia and resistant to elevated temperatures, can accumulate under mass-rearing conditions and lead to the loss of the sexing properties and the breakdown of the genetic sexing strain [17,18].

It is also important to note that the GSS strains tested in the present study: (a) are products of two different translocations (VIENNA 7 and VIENNA 8), (b) may include additional markers such as Sr^2^ [24], (c) may have been (re)constructed at different time points and (d) may have been introgressed into local genetic background depending on the region of SIT applications. These factors may have affected their recombination rates due to the distance between the translocation break point and the selectable markers [17] and their response to elevated temperatures [18]. Our results showed significant temperature sensitivity at temperatures lower than 34 °C and 35 °C resulting in female lethality and reduced male recovery. This is important because, in the context of operational SIT programmes, reduced male recovery rates would increase the cost of the applications. SIT facilities may decide to change the strain opting for a strain with higher fitness at 25 °C and improving the overall performance of their strain by enriching it with a local genetic background. In addition, if complete female lethality can be achieved at a lower temperature, the recovered males will have experienced less stress and may be of higher biological quality. Bottlenecks, long-term inbreeding, accumulation of spontaneous mutations, and introgression into local genetic backgrounds may have played a role in modulating the tsl phenotype, the rearing efficiency, and the characteristics of the genetic sexing strains. Several studies have reported that prolonged mass-rearing leads to laboratory adaptation, which sequentially can result in loss of genetic diversity and significant changes in alleles [29,30,31,32]. These alterations can affect various phenotypes and life-history traits. Therefore, they should be continuously monitored to verify that genetic diversity, biological quality, and competitiveness are maintained and that the production of high-quality sterile insects for SIT applications is assured [33,34]. Towards this goal, periodic refreshments of mass-reared colonies with wild or partially inbred material have been proven to greatly value mass-rearing facilities [29,31,35]. Infusion of the mass-reared colony with “fresh” flies can limit genetic bottlenecks and maintain the genetic diversity and biological quality of the mass-reared insects.

Analysis of four different *wp tsl* strains indicated that they fully expressed the temperature-sensitive lethal phenotype. In contrast, out of the three inversion lines studied, only one of them (D53-1) behaved like a typical *tsl* mutant strain; however, the other two lines (having the same origin but kept for a long time in separate rearing rooms) demonstrated a significant number of survivors at both 34 °C and 35 °C. All three strains contain the D53 inversion, which covers parts of chromosome 5 [In(5)50C;59C]. Chromosomal inversions are known suppressors of genetic recombination, and therefore, we would expect that the D53 inversion, which resides in the *wp tsl* genomic region, would suppress recombination in that region [17]. The left breakpoint of the inversion is located between the *wp* and *tsl* loci, being closer to the *wp* locus [19,36]. Therefore, recombination phenomena could be possible as the *tsl* locus is outside the borders of the D53 inversion. A revertant mutation at the *tsl* gene combined with genetic recombination could explain the presence of survivors at elevated temperatures, thus creating *wp tsl^+^* individuals. Mutation(s) on secondary genetic loci might also affect the expression of the tsl phenotype. The latter may require exposure to higher temperatures to achieve complete lethality, as recently shown for another medfly strain carrying the *tsl* mutation [37]. 

Differences were detected among the tested wild-type strains at elevated temperatures 34 °C and 35 °C. For example, the hatching rate of the “Benakeion Volos” strain was low at both temperatures compared to the wild-type strain EgII FF26 suggesting that temperature-sensitive lethal alleles (not necessary of the same *tsl* locus) may be present in this strain, and they could be potentially harnessed for the establishment of novel temperature-sensitive lethal strains. On the other hand, the “Seibersdorf” and “Argentina” strains exhibited high eclosion rates, suggesting they are more tolerant at high temperatures. This property could be helpful for SIT applications in regions with a warmer climate.

Moreover, climate change increases the need to characterize such mutations and is not to be underestimated [38]. In combination with the invasive character of *C. capitata,* climate change favoured the shift of its geographic range into new areas [39,40]. On the other hand, climate change can severely affect an AW-IPM programme because temperature changes can influence the sterile males and male mating competitiveness. Rising temperatures could lead to the death of sterile males released during SIT. However, it should be noted that *Ceratitis capitata* can adapt to such stressful conditions [41,42]. It has been observed that virgin sexually mature males prefer a warmer temperature than females (±1.7 °C), also keeping an optimal mating propensity [41]. Since the global surface annual temperature has increased at an average rate of 0.1 °C, almost double compared to 20 years ago, and increases of 1.5 °C and 2-4 °C are expected by 2050 and 2100, respectively [43], *C. capitata* may still have room for adaptation over the next years. This may speed up the life cycle, increase the number of generations per year, and expand its geographic range. In addition, it is essential to note that climate change has also resulted in the unpredictable appearance and frequency of weather phenomena such as storms, heavy rains, and heat and cold waves. Those may impact IPM programmes with an SIT component, especially during the release of sterile males, since such phenomena may affect their longevity, flight ability, and mating performance.

## 5. Conclusions

The temperature-sensitive lethal tests on twenty-seven *Ceratitis capitata* strains resulted in significant differences. The homozygous lethal *tsl* alleles in mutant strains and GSS females trigger embryonic lethality and subsequent reduction in pupal recovery and adult emergence rates when embryos are subjected to elevated temperatures. However, variability was observed in the lethality levels, which could be due to novel mutations resulting in revertant alleles or changes in the genetic background, which might influence the expression levels and/or the penetrance of the tsl phenotype. Variability was observed among the wild-type strains suggesting that some may be more sensitive to elevated temperatures than others. In addition, some of the *tsl* mutant strains may express the tsl phenotype at lower than usual temperatures (less than 34 °C). The results of the present study indicate that TSLTs should frequently be carried out with strains used in SIT-related projects, both in research labs and operational programmes. In addition, the thermal tolerance of GSS males used in SIT field applications should be tested because high temperatures occur more frequently due to climate change. 

## Figures and Tables

**Table 1:** *Ceratitis capitata* strains used in the present study.

	Strain	Group
1	EgII FF21 ^a^	wild-type
2	EgII FF26 ^a^	wild-type
3	Benakeion Volos FF26	wild-type
4	Seibersdorf (SEIB) FF26	wild-type
5	Argentina FF26	wild-type
6	Benakeion TR 34 FF26 ^b^	wild-type
7	Benakeion TR 35 FF26 ^b^	wild-type
8	Benakeion TR 34S FF26 ^c^	wild-type
9	Benakeion TR 35S FF26 ^c^	wild-type
10	VIENNA 8 2010 FF26 ^d^	GSS
11	VIENNA 8 2018 FF26 ^d^	GSS
12	VIENNA 8 2019 FF26 ^d^	GSS
13	VIENNA 8 Sr^2^ FF26 ^e^	GSS
14	VIENNA 8 “El Pino” FF26	GSS
15	VIENNA 8 Israel FF26	GSS
16	VIENNA 8 Argentina FF26	GSS
17	VIENNA 7 2017 FF26 ^d^	GSS
18	VIENNA 7 2018 FF26 ^d^	GSS
19	VIENNA 7 2019 FF26 ^d^	GSS
20	VIENNA 7 2020 FF26 ^d^	GSS
21	*wp tsl* FF21 ^a^	mutant
22	*wp tsl* FF26 ^a^	mutant
23	*wp tsl* (EgII) FF21 ^a^	mutant
24	*wp tsl* (EgII) FF26 ^a^	mutant
25	D53-3-28 FF21 ^a^	mutant
26	D53-3-28 FF26 ^a^	mutant
27	D53-1 FF26	mutant

^a^ Strains were kept as parallel cultures in two rooms, FF21 and FF26, at the IPCL under the same rearing conditions for backup purposes. ^b^ The Benakeion TR 34 and Benakeion TR 35 strains were established with Benakeion strain survivors of TSLT conducted at 34 °C and 35 °C, respectively. ^c^ The Benakeion TR 34S and Benakeion TR 35S strains were established with Benakeion TR and Benakeion TR 35 strain survivors of TSLT performed at the next generation at 34 °C and 35 °C, respectively. ^d^ VIENNA 7 and VIENNA 8 GSS, generated in the respective years.^e^ VIENNA 8 GSS, carrying the *Sr^2^* dominant mutation, a homozygous lethal mutation that leads to the expression of a third stripe on the fly’s abdomen [22].

## Data Availability

All data generated or analyzed during this study are included in the manuscript.

## Supplementary Materials

The following supporting information can be downloaded at: https://www.mdpi.com/article/10.3390/insects13100943/s1, Table S1: Egg hatching, pupal recovery, and adult emergence rates at 25 °C, 34 °C and 35 °C for twenty-seven *Ceratitis capitata* strains. Table S2: Pairwise comparisons of egg hatching at 25 °C, 34 °C and 35 °C for twenty-seven *Ceratitis capitata* strains. Table S3: Pairwise comparisons of pupal recovery at 25 °C, 34 °C and 35 °C for twenty-seven *Ceratitis capitata* strains. Table S4: Pairwise comparisons of adult recovery at 25 °C, 34 °C and 35 °C for twenty-seven *Ceratitis capitata* strains. Table S5: Temperature pairwise comparisons of egg hatching at 25 °C, 31 °C, 32 °C, 33 °C, 34 °C and 35 °C for GSS and *tsl* mutant *Ceratitis capitata* strains.
