# Supplementary material for: Temperature Sensitivity of Wild-Type, Mutant and Genetic Sexing Strains of Ceratitis capitata
Source: Insects. 2022 Oct 18;13(10):943. doi: 10.3390/insects13100943 (PMC9604331; doi:10.3390/insects13100943)
Supplement: Supplementary file 1 [file insects-13-00943-s001.zip › insects-1861677-supplementary.pdf]

Supplementary Material

Table S1: Egg hatching, pupal recovery, and adult emergence rates at 25 °C, 34 °C and 35 °C for twenty-seven *Ceratitis capitata* strains.

Strain	Temperature (°C)	Egg hatching* (%)	Pupal recovery* (%)	Adult emergence* (%)
EgII FF21	25	93.89	86.39	90.96
	34	67.78	52.46	92.50
	35	54.78	22.52	95.50
EgII FF26	25	98.00	89.00	87.52
	34	83.67	72.64	89.76
	35	44.44	32.25	84.50
Benakeion Volos FF26	25	87.33	86.01	93.64
	34	29.89	35.32	93.68
	35	14.22	12.50	93.75
Seibersdorf (SEIB) FF26	25	89.78	93.81	99.47
	34	67.67	79.15	98.34
	35	59.22	47.09	94.82
Argentina FF26	25	89.67	88.85	97.21
	34	67.11	76.99	91.83
	35	43.78	49.75	97.96
Benakeion TR 34 FF26	25	93.89	86.39	90.96
	34	67.78	52.46	92.50
	35	54.78	22.52	95.50
Benakeion TR 35 FF26	25	92.89	79.31	94.27
	34	62.78	48.32	90.84
	35	48.56	26.32	89.57
Benakeion TR 34 S FF26	25	85.11	89.69	90.39
	34	55.89	71.17	98.32
	35	25.89	27.04	77.78
Benakeion TR 35 S FF26	25	85.44	88.43	90.15
	34	61.78	65.65	95.89
	35	33.00	19.87	94.92
VIENNA 8 2010 FF26	25	65.22	85.35	76.25
	31	44.22	66.83	72.18
	32	39.56	64.33	92.14
	33	29.44	72.08	87.43
	34	21.90	59.82	89.31
	35	19.89	49.72	76.40
VIENNA 8 2018 FF26	25	72.78	68.85	71.84
	31	43.89	48.10	78.42
	32	45.33	50.49	76.70
	33	25.44	60.26	88.41
	34	21.40	50.47	79.63
	35	9.33	52.38	81.82
	25	79.22	64.66	89.59

VIENNA 8 2019 FF26	31	68.33	67.32	90.34
	32	67.44	70.68	88.11
	33	56.89	66.99	91.84
	34	22.00	56.36	84.68
	35	4.33	25.64	100.00
VIENNA 8 Sr^2 FF26	25	76.44	89.53	78.41
	31	58.33	72.76	78.80
	32	45.00	71.85	82.82
	33	35.00	74.92	83.47
	34	16.60	73.49	89.34
	35	13.33	56.67	77.94
VIENNA 8 Guatemala "El Pino" FF26	25	82.56	74.56	85.02
	31	69.67	70.33	83.22
	32	73.89	66.62	87.81
	33	56.22	67.39	85.34
	34	26.50	44.53	88.98
	35	12.89	24.14	89.29
VIENNA 8 Israel FF26	25	92.00	88.53	81.31
	31	89.67	75.71	78.56
	32	88.89	80.25	75.70
	33	67.11	69.21	86.36
	34	37.20	76.08	89.40
	35	28.44	66.02	84.62
VIENNA 8 Argentina FF26	25	91.00	76.56	88.52
	31	86.89	74.42	87.97
	32	89.89	72.81	91.51
	33	84.89	66.36	94.67
	34	68.20	52.49	91.62
	35	40.22	37.85	91.24
VIENNA 7 2017 FF26	25	76.11	81.61	72.45
	31	46.56	69.93	78.84
	32	58.00	67.05	78.29
	33	31.89	74.22	85.92
	34	19.20	60.94	90.60
	35	6.67	45.00	77.78
VIENNA 7 2018 FF26	25	72.78	69.31	68.72
	31	60.67	58.06	77.92
	32	59.56	56.53	74.59
	33	37.00	66.67	83.33
	34	25.80	54.65	80.14
	35	8.56	36.36	85.71
VIENNA 7 2019 FF26	25	82.89	63.00	76.81
	31	72.78	56.64	87.60
	32	67.89	64.48	85.53
	33	69.33	54.33	86.73
	34	26.30	64.64	91.76
	35	10.44	28.72	77.78
VIENNA 7 2020 FF26	25	81.22	78.11	87.39
	31	60.78	57.59	85.40

	32	54.22	67.83	76.74
	33	29.56	81.20	95.37
	34	16.80	64.88	97.25
	35	5.89	33.96	94.44
wp/tsl FF21	25	91.33	86.25	79.13
	31	79.33	50.70	48.62
	32	28.78	50.97	57.58
	33	8.89	26.25	28.57
	34	1.00	10.00	0.00
	35	1.00	0.00	0.00
wp/tsl FF26	25	86.22	83.63	95.07
	31	74.78	69.99	63.91
	32	75.44	68.48	48.17
	33	20.44	39.13	88.89
	34	2.30	26.09	50.00
	35	1.22	0.00	0.00
wp/tsl (EgII) FF21	25	94.33	94.94	93.30
	31	75.78	75.37	51.75
	32	78.44	79.32	54.64
	33	10.11	25.27	39.13
	34	1.00	0.00	0.00
	35	1.00	0.00	0.00
wp/tsl (EgII) FF26	25	89.56	88.96	95.82
	31	80.56	76.83	72.17
	32	82.00	80.35	74.03
	33	27.56	62.50	80.00
	34	1.10	18.18	50.00
	35	1.56	7.14	100.00
D53-3-28 FF21	25	95.78	97.33	95.47
	31	88.33	90.94	96.68
	32	88.56	88.83	96.05
	33	79.56	84.08	92.19
	34	42.10	54.39	89.08
	35	17.67	32.08	90.20
D53-3-28 FF26	25	96.33	93.08	95.17
	31	91.56	91.99	91.42
	32	92.33	93.14	93.02
	33	82.44	73.99	89.07
	34	51.70	54.35	85.77
	35	26.33	37.97	78.89
D53-1 FF26	25	95.11	94.39	89.36
	31	81.33	67.21	89.43
	32	84.33	70.36	91.39
	33	17.11	23.38	58.33
	34	1.00	0.00	0.00
	35	1.00	0.00	0.00

* The values are presented as mean ± standard deviation.

Table S2: Pairwise comparisons of egg hatching at 25 °C, 34 °C and 35 °C for twenty-seven *Ceratitis capitata* strains.

Strain	Temperature [°C] pairwise comparisons	Estimate	Standard Error	df	z ratio	*p* value
EgII_FF21	25 - 34	0.326	0.0724	Inf	4.503	<.0001
	25 - 35	0.539	0.0929	Inf	5.801	<.0001
	34 - 35	0.213	0.1123	Inf	1.897	0.0579
EgII_FF26	25 - 34	0.158	0.0507	Inf	3.121	0.0018
	25 - 35	0.791	0.123	Inf	6.43	<.0001
	34 - 35	0.633	0.1312	Inf	4.823	<.0001
Benakeion Volos FF26	25 - 34	2.783	0.124	Inf	22.468	<.0001
	25 - 35	3.728	0.138	Inf	26.936	<.0001
	34 - 35	0.944	0.12	Inf	7.867	<.0001
Seibersdorf (SEIB) FF26	25 - 34	0.283	0.0356	Inf	7.95	<.0001
	25 - 35	0.416	0.0414	Inf	10.046	<.0001
	34 - 35	0.133	0.0499	Inf	2.67	0.0076
Argentina FF26	25 - 34	0.29	0.0565	Inf	5.126	<.0001
	25 - 35	0.717	0.0859	Inf	8.342	<.0001
	34 - 35	0.427	0.0968	Inf	4.415	<.0001
Benakeion TR 34 FF26	25 - 34	0.326	0.0724	Inf	4.503	<.0001
	25 - 35	0.539	0.0929	Inf	5.801	<.0001
	34 - 35	0.213	0.1123	Inf	1.897	0.0579
Benakeion TR 35 FF26	25 - 34	0.392	0.072	Inf	5.442	<.0001
	25 - 35	0.649	0.0938	Inf	6.917	<.0001
	34 - 35	0.257	0.1131	Inf	2.271	0.0231
Benakeion TR 34 S FF26	25 - 34	0.421	0.0546	Inf	7.703	<.0001
	25 - 35	1.19	0.0969	Inf	12.281	<.0001
	34 - 35	0.77	0.1063	Inf	7.242	<.0001
Benakeion TR 35 S FF26	25 - 34	0.324	0.0559	Inf	5.807	<.0001
	25 - 35	0.951	0.0933	Inf	10.2	<.0001
	34 - 35	0.627	0.1023	Inf	6.127	<.0001
VIENNA 8 2010 FF26	25 - 34	1.9605	0.108	Inf	18.185	<.0001
	25 - 35	2.0221	0.109	Inf	18.558	<.0001
	34 - 35	0.0616	0.117	Inf	0.526	0.5986
VIENNA 8 2018 FF26	25 - 34	1.222	0.129	Inf	9.468	<.0001
	25 - 35	2.066	0.205	Inf	10.062	<.0001
	34 - 35	0.844	0.236	Inf	3.575	0.0004
VIENNA 8 2019 FF26	25 - 34	1.18	0.106	Inf	11.103	<.0001
	25 - 35	2.96	0.281	Inf	10.522	<.0001
	34 - 35	1.78	0.298	Inf	5.974	<.0001
VIENNA 8 Sr^2 FF26	25 - 34	1.428	0.231	Inf	6.192	<.0001
	25 - 35	1.746	0.276	Inf	6.334	<.0001
	34 - 35	0.318	0.35	Inf	0.911	0.3625
VIENNA 8 Guatemala "El Pino" FF26	25 - 34	1.1	0.108	Inf	10.17	<.0001
	25 - 35	1.86	0.169	Inf	10.988	<.0001
	34 - 35	0.76	0.196	Inf	3.874	0.0001

VIENNA 8 Israel FF26	25 - 34	0.853	0.0591	Inf	14.423	<.0001
	25 - 35	1.174	0.0745	Inf	15.747	<.0001
	34 - 35	0.321	0.0932	Inf	3.449	0.0006
VIENNA 8 Argentina FF26	25 - 34	0.234	0.0475	Inf	4.935	<.0001
	25 - 35	0.816	0.0855	Inf	9.544	<.0001
	34 - 35	0.582	0.093	Inf	6.258	<.0001
VIENNA 7 2017 FF26	25 - 34	1.28	0.16	Inf	7.986	<.0001
	25 - 35	2.47	0.307	Inf	8.044	<.0001
	34 - 35	1.19	0.34	Inf	3.502	0.0005
VIENNA 7 2018 FF26	25 - 34	0.996	0.132	Inf	7.557	<.0001
	25 - 35	2.167	0.253	Inf	8.577	<.0001
	34 - 35	1.171	0.277	Inf	4.222	<.0001
VIENNA 7 2019 FF26	25 - 34	1.08	0.122	Inf	8.842	<.0001
	25 - 35	2.1	0.222	Inf	9.482	<.0001
	34 - 35	1.02	0.249	Inf	4.108	<.0001
VIENNA 7 2020 FF26	25 - 34	1.53	0.111	Inf	13.781	<.0001
	25 - 35	2.62	0.202	Inf	13.015	<.0001
	34 - 35	1.09	0.228	Inf	4.797	<.0001
wp/tsl FF21	25 - 34	42.2	61.06	Inf	2.583	0.0098
	25 - 35	42.2	61.06	Inf	2.583	0.0098
	34 - 35	1	1.18	Inf	0	1
wp/tsl FF26	25 - 34	25	31.96	Inf	2.522	0.0117
	25 - 35	25	31.96	Inf	2.522	0.0117
	34 - 35	1	1.18	Inf	0	1
wp/tsl (EgII) FF21	25 - 34	56.255	84.191	Inf	2.693	0.0071
	25 - 35	42.154	61.06	Inf	2.583	0.0098
	34 - 35	0.749	0.927	Inf	-0.233	0.8156
wp/tsl (EgII) FF26	25 - 34	34.3	47.06	Inf	2.577	0.01
	25 - 35	34.3	47.06	Inf	2.577	0.01
	34 - 35	1	1.18	Inf	0	1
D53-3-28 FF21	25 - 34	26.51	47.38	Inf	1.834	0.0667
	25 - 35	105.72	198.1	Inf	2.487	0.0129
	34 - 35	3.99	4.39	Inf	1.257	0.2088
D53-3-28 FF26	25 - 34	20.87	38.59	Inf	1.643	0.1004
	25 - 35	69.14	130.18	Inf	2.25	0.0245
	34 - 35	3.31	3.35	Inf	1.186	0.2356
D53-1 FF26	25 - 34	77.8	136.66	Inf	2.48	0.0132
	25 - 35	77.8	136.66	Inf	2.48	0.0132
	34 - 35	1	1.18	Inf	0	1

* Inf = Infinite degrees of freedom. Z test used for pairwise comparison is not affected by the number of observations.

Table S3: Pairwise comparisons of pupal recovery at 25 °C, 34 °C and 35 °C for twenty-seven *Ceratitis capitata* strains.

Strain	Temperature [°C] pairwise comparisons	Estimate	Standard Error	df	z ratio	*p* value
EgII_FF21	25 - 34	0.499	0.121	Inf	4.13	<.0001
	25 - 35	1.345	0.25	Inf	5.378	<.0001
	34 - 35	0.846	0.272	Inf	3.112	0.0019
EgII_FF26	25 - 34	0.203	0.0632	Inf	3.215	0.0013
	25 - 35	1.015	0.1833	Inf	5.537	<.0001
	34 - 35	0.812	0.1894	Inf	4.288	<.0001
Benakeion Volos FF26	25 - 34	0.89	0.123	Inf	7.209	<.0001
	25 - 35	1.93	0.345	Inf	5.585	<.0001
	34 - 35	1.04	0.366	Inf	2.841	0.0045
Seibersdorf (SEIB) FF26	25 - 34	0.17	0.0467	Inf	3.639	0.0003
	25 - 35	0.689	0.0964	Inf	7.149	<.0001
	34 - 35	0.519	0.1038	Inf	5	<.0001
Argentina FF26	25 - 34	0.153	0.0615	Inf	2.487	0.0129
	25 - 35	0.59	0.1268	Inf	4.65	<.0001
	34 - 35	0.437	0.1348	Inf	3.239	0.0012
Benakeion TR 34 FF26	25 - 34	0.511	0.123	Inf	4.164	<.0001
	25 - 35	1.345	0.251	Inf	5.35	<.0001
	34 - 35	0.833	0.274	Inf	3.043	0.0023
Benakeion TR 35 FF26	25 - 34	1.411	0.285	Inf	4.95	<.0001
	25 - 35	2.373	0.328	Inf	7.227	<.0001
	34 - 35	0.962	0.327	Inf	2.946	0.0032
Benakeion TR 34 S FF26	25 - 34	0.231	0.0595	Inf	3.885	0.0001
	25 - 35	1.199	0.2086	Inf	5.749	<.0001
	34 - 35	0.968	0.2143	Inf	4.516	<.0001
Benakeion TR 35 S FF26	25 - 34	0.298	0.0609	Inf	4.888	<.0001
	25 - 35	1.493	0.2144	Inf	6.965	<.0001
	34 - 35	1.195	0.2203	Inf	5.426	<.0001
VIENNA 8 2010 FF26	25 - 34	0.342	0.0986	Inf	3.467	0.0005
	25 - 35	0.54	0.1242	Inf	4.351	<.0001
	34 - 35	0.199	0.1537	Inf	1.292	0.1965
VIENNA 8 2018 FF26	25 - 34	0.379	0.155	Inf	2.441	0.0147
	25 - 35	0.261	0.205	Inf	1.274	0.2026
	34 - 35	-0.117	0.247	Inf	-0.474	0.6354
VIENNA 8 2019 FF26	25 - 34	0.338	0.157	Inf	2.147	0.0318
	25 - 35	1.597	0.378	Inf	4.221	<.0001
	34 - 35	1.26	0.394	Inf	3.193	0.0014
VIENNA 8 Sr^2 FF26	25 - 34	0.191	0.0684	Inf	2.8	0.0051
	25 - 35	0.457	0.1151	Inf	3.973	0.0001
	34 - 35	0.266	0.1313	Inf	2.026	0.0427
VIENNA 8 Guatemala "El Pino" FF26	25 - 34	1.237	0.153	Inf	8.099	<.0001
	25 - 35	2.221	0.233	Inf	9.54	<.0001
	34 - 35	0.983	0.252	Inf	3.909	0.0001
	25 - 34	0.146	0.0481	Inf	3.036	0.0024

VIENNA 8 Israel FF26	25 - 35	0.293	0.0699	Inf	4.195	<.0001
	34 - 35	0.147	0.0806	Inf	1.826	0.0679
VIENNA 8 Argentina FF26	25 - 34	1.047	0.169	Inf	6.199	<.0001
	25 - 35	1.68	0.202	Inf	8.324	<.0001
	34 - 35	0.632	0.198	Inf	3.185	0.0014
VIENNA 7 2017	25 - 34	1.032	0.178	Inf	5.787	<.0001
	25 - 35	1.628	0.281	Inf	5.791	<.0001
	34 - 35	0.596	0.302	Inf	1.973	0.0485
VIENNA 7 2018	25 - 34	0.217	0.0956	Inf	2.27	0.0232
	25 - 35	0.655	0.2376	Inf	2.758	0.0058
	34 - 35	0.438	0.2499	Inf	1.753	0.0797
VIENNA 7 2019	25 - 34	-0.0644	0.101	Inf	-0.636	0.5251
	25 - 35	0.7276	0.311	Inf	2.338	0.0194
	34 - 35	0.792	0.318	Inf	2.49	0.0128
VIENNA 7 2020 FF26	25 - 34	0.14	0.0818	Inf	1.708	0.0876
	25 - 35	0.833	0.272	Inf	3.062	0.0022
	34 - 35	0.693	0.2813	Inf	2.464	0.0137
wp/tsl FF21	25 - 34	14.8	17.99	Inf	2.22	0.0264
	25 - 35	14.8	17.99	Inf	2.22	0.0264
	34 - 35	1	1.03	Inf	0	1
wp/tsl FF26	25 - 34	11.9	13.73	Inf	2.139	0.0325
	25 - 35	11.9	13.73	Inf	2.139	0.0325
	34 - 35	1	1.03	Inf	0	1
wp/tsl (EgII) FF21	25 - 34	44.4	75.15	Inf	2.239	0.0252
	25 - 35	44.4	75.15	Inf	2.239	0.0252
	34 - 35	1	1.03	Inf	0	1
wp/tsl (EgII) FF26	25 - 34	18.9	24.4	Inf	2.278	0.0227
	25 - 35	18.9	24.4	Inf	2.278	0.0227
	34 - 35	1	1.03	Inf	0	1
D53-3-28 FF21	25 - 34	29.86	64.79	Inf	1.566	0.1174
	25 - 35	77.34	168.89	Inf	1.991	0.0465
	34 - 35	2.59	2.54	Inf	0.971	0.3314
D53-3-28 FF26	25 - 34	10.66	15.45	Inf	1.631	0.1028
	25 - 35	20.73	30.22	Inf	2.079	0.0376
	34 - 35	1.95	1.86	Inf	0.694	0.4876
D53-1 FF26	25 - 34	39.5	64.25	Inf	2.263	0.0236
	25 - 35	39.5	64.25	Inf	2.263	0.0236
	34 - 35	1	1.03	Inf	0	1

* Inf = Infinite degrees of freedom. Z test used for pairwise comparison is not affected by the number of observations.

Table S4: Pairwise comparisons of adult recovery at 25 °C, 34 °C and 35 °C for twenty-seven *Ceratitis capitata* strains.

Strain	Temperature [°C] pairwise comparisons	Estimate	Standard Error	df	z ratio	*p* value
EgII_FF21	25 - 34	-0.204	0.488	Inf	-0.418	0.6762
	25 - 35	-0.745	0.933	Inf	-0.799	0.4246
	34 - 35	-0.542	0.99	Inf	-0.547	0.5844
EgII_FF26	25 - 34	-0.224	0.569	Inf	-0.393	0.6943
	25 - 35	0.252	0.853	Inf	0.295	0.7679
	34 - 35	0.475	0.901	Inf	0.528	0.5977
Benakeion Volos	25 - 34	-0.00761	0.679	Inf	-0.011	0.9911
	25 - 35	-0.01878	1.575	Inf	-0.012	0.9905
	34 - 35	-0.01117	1.681	Inf	-0.007	0.9947
Seibersdorf (SEIB) FF26	25 - 34	1.16	1.137	Inf	1.018	0.3087
	25 - 35	2.33	1.066	Inf	2.188	0.0286
	34 - 35	1.17	0.843	Inf	1.393	0.1637
Argentina FF26	25 - 34	0.912	0.627	Inf	1.454	0.1461
	25 - 35	-0.54	1.29	Inf	-0.419	0.6753
	34 - 35	-1.452	1.262	Inf	-1.15	0.25
Benakeion TR 34 FF26	25 - 34	-0.19	0.487	Inf	-0.391	0.6961
	25 - 35	-0.745	0.931	Inf	-0.801	0.4234
	34 - 35	-0.555	0.988	Inf	-0.562	0.574
Benakeion TR 35 FF26	25 - 34	0.506	0.381	Inf	1.328	0.1843
	25 - 35	0.65	0.494	Inf	1.317	0.1879
	34 - 35	0.145	0.526	Inf	0.275	0.7831
Benakeion TR 34 S FF26	25 - 34	-1.83	0.971	Inf	-1.885	0.0594
	25 - 35	0.989	0.741	Inf	1.334	0.1822
	34 - 35	2.819	1.15	Inf	2.452	0.0142
Benakeion TR 35 S FF26	25 - 34	-0.936	1.05	Inf	-0.894	0.3713
	25 - 35	-0.713	2.16	Inf	-0.329	0.7418
	34 - 35	0.223	2.32	Inf	0.096	0.9232
VIENNA 8 2010 FF26	25 - 34	-0.88387	0.479	Inf -	1.845	0.065
	25 - 35	-0.00869	0.415	Inf	-0.021	0.9833
	34 - 35	0.87519	0.591	Inf	1.48	0.1389
VIENNA 8 2018 FF26	25 - 34	-0.054	0.11	Inf	-0.49	0.6242
	25 - 35	-0.1301	0.128	Inf	-1.015	0.3103
	34 - 35	-0.0761	0.154	Inf	-0.494	0.6215
VIENNA 8 2019 FF26	25 - 34	0.443	0.434	Inf	1.019	0.308
	25 - 35	-16.675	3492.268	Inf	-0.005	0.9962
	34 - 35	-17.117	3492.268	Inf	-0.005	0.9961
VIENNA 8 Sr^2 FF26	25 - 34	-0.8367	0.384	Inf	-2.181	0.0292
	25 - 35	0.0274	0.383	Inf	0.072	0.9428
	34 - 35	0.8642	0.514	Inf	1.682	0.0926
VIENNA 8 Guatemala "El Pino" FF26	25 - 34	-0.404	0.385	Inf	-1.049	0.2942
	25 - 35	-0.3842	0.732	Inf	-0.525	0.5996
	34 - 35	0.0198	0.803	Inf	0.025	0.9803
	25 - 34	-0.686	0.366	Inf	-1.875	0.0608

VIENNA 8 Israel FF26	25 - 35	-0.234	0.387	Inf	-0.607	0.5441
	34 - 35	0.452	0.484	Inf	0.934	0.3503
VIENNA 8 Argentina FF26	25 - 34	-0.0407	0.0467	Inf	-0.871	0.3835
	25 - 35	-0.0303	0.0663	Inf	-0.457	0.6474
	34 - 35	0.0104	0.0676	Inf	0.154	0.8776
VIENNA 7 2017 FF26	25 - 34	-1.299	0.331	Inf	-3.928	0.0001
	25 - 35	-0.286	0.472	Inf	-0.605	0.5452
	34 - 35	1.013	0.561	Inf	1.806	0.071
VIENNA 7 2018 FF26	25 - 34	-0.553	0.242	Inf	-2.288	0.0221
	25 - 35	-0.781	0.516	Inf	-1.514	0.1301
	34 - 35	-0.228	0.551	Inf	-0.414	0.6789
VIENNA 7 2019 FF26	25 - 34	-1.2133	0.356	Inf	-3.411	0.0006
	25 - 35	0.0989	0.534	Inf	0.185	0.8531
	34 - 35	1.3122	0.615	Inf	2.134	0.0328
VIENNA 7 2020 FF26	25 - 34	-1.619	0.842	Inf	-1.924	0.0544
	25 - 35	-0.897	1.457	Inf	-0.616	0.538
	34 - 35	0.722	1.664	Inf	0.434	0.6643
wp/tsl FF21	25 - 34	NA				
	25 - 35					
	34 - 35					
wp/tsl FF26	25 - 34	2.96	1.93	Inf	1.533	0.1254
	25 - 35	NA				
	34 - 35					
wp/tsl (EgII) FF21	25 - 34	NA				
	25 - 35					
	34 - 35					
wp/tsl (EgII) FF26	25 - 34	3.13	2.98	Inf	1.051	0.2935
	25 - 35	-13.43	5013.62	Inf	-0.003	0.9979
	34 - 35	-16.57	5013.62	Inf	-0.003	0.9974
D53-3-28 FF21	25 - 34	0.0693	0.0367	Inf	1.886	0.0593
	25 - 35	0.0568	0.0706	Inf	0.805	0.4208
	34 - 35	-0.0124	0.0779	Inf	-0.159	0.8734
D53-3-28 FF26	25 - 34	0.0988	0.0393	Inf	2.516	0.0119
	25 - 35	0.19	0.0847	Inf	2.244	0.0248
	34 - 35	0.0911	0.0918	Inf	0.993	0.3208
D53-1 FF26	25 - 34	NA				
	25 - 35					
	34 - 35					

* Inf = Infinite degrees of freedom. Z test used for pairwise comparison is not affected by the number of observations.

Table S5: Temperature pairwise comparisons of egg hatching at 25 °C, 31 °C, 32 °C, 33 °C, 34 °C and 35 °C for GSS and *tsl* mutant *Ceratitis capitata* strains.

Strain	Temperature [°C] pairwise comparisons	Estimate	Standard Error	df	z ratio	*p* value
VIENNA 8 2010 FF26	25 - 31	0.3886	0.0568	Inf	6.847	<.0001
	31 - 32	0.1115	0.0708	Inf	1.576	0.115
	32 - 33	0.2952	0.0839	Inf	3.517	0.0004
	33 - 34	0.3433	0.1053	Inf	3.259	0.0011
	34 - 35	0.0491	0.1184	Inf	0.414	0.6787
VIENNA 8 2018 FF26	25 - 31	0.5057	0.0847	Inf	5.97	<.0001
	31 - 32	-0.0324	0.1039	Inf	-0.312	0.7552
	32 - 33	0.5775	0.134	Inf	4.309	<.0001
	33 - 34	0.171	0.1692	Inf	1.011	0.3122
	34 - 35	0.8319	0.241	Inf	3.451	0.0006
VIENNA 8 2019 FF26	25 - 31	0.1479	0.0512	Inf	2.888	0.0039
	31 - 32	0.0131	0.0585	Inf	0.224	0.8228
	32 - 33	0.1702	0.0669	Inf	2.543	0.011
	33 - 34	0.8493	0.1182	Inf	7.186	<.0001
	34 - 35	1.7255	0.3016	Inf	5.721	<.0001
VIENNA 8 Sr^2 FF26	25 - 31	0.27	0.0907	Inf	2.98	0.0029
	31 - 32	0.26	0.1249	Inf	2.078	0.0377
	32 - 33	0.251	0.1575	Inf	1.596	0.1105
	33 - 34	0.647	0.2254	Inf	2.868	0.0041
	34 - 35	0.318	0.297	Inf	1.072	0.2836
VIENNA 8 Guatemala "El Pino" FF26	25 - 31	0.1697	0.0541	Inf	3.138	0.0017
	31 - 32	-0.0588	0.0597	Inf	-0.985	0.3246
	32 - 33	0.2733	0.0716	Inf	3.818	0.0001
	33 - 34	0.7131	0.1242	Inf	5.743	<.0001
	34 - 35	0.7598	0.2061	Inf	3.687	0.0002
VIENNA 8 Israel FF26	25 - 31	0.02569	0.0187	Inf	1.377	0.1684
	31 - 32	0.00871	0.0203	Inf	0.428	0.6683
	32 - 33	0.28104	0.0325	Inf	8.638	<.0001
	33 - 34	0.53711	0.0592	Inf	9.066	<.0001
	34 - 35	0.32129	0.0836	Inf	3.841	0.0001
VIENNA 8 Argentina FF26	25 - 31	0.0462	0.028	Inf	1.652	0.0984
	31 - 32	-0.0339	0.0287	Inf	-1.182	0.2374
	32 - 33	0.0572	0.0302	Inf	1.897	0.0578
	33 - 34	0.1647	0.0421	Inf	3.907	0.0001
	34 - 35	0.5822	0.0766	Inf	7.596	<.0001
VIENNA 7 2017 FF26	25 - 31	0.492	0.0788	Inf	6.237	<.0001
	31 - 32	-0.22	0.0892	Inf	-2.464	0.0137
	32 - 33	0.598	0.1102	Inf	5.426	<.0001
	33 - 34	0.407	0.1576	Inf	2.583	0.0098
	34 - 35	1.158	0.2743	Inf	4.221	<.0001
VIENNA 7 2018 FF26	25 - 31	0.182	0.059	Inf	3.086	0.002
	31 - 32	0.0185	0.0672	Inf	0.275	0.7833
	32 - 33	0.476	0.09	Inf	5.287	<.0001

	33 - 34	0.3192	0.1227	Inf	2.602	0.0093
	34 - 35	1.1451	0.2136	Inf	5.361	<.0001
VIENNA 7 2019 FF26	25 - 31	0.1301	0.0466	Inf	2.794	0.0052
	31 - 32	0.0695	0.0562	Inf	1.237	0.2162
	32 - 33	-0.0211	0.0585	Inf	-0.36	0.7187
	33 - 34	0.9028	0.1058	Inf	8.531	<.0001
	34 - 35	0.9901	0.2039	Inf	4.857	<.0001
VIENNA 7 2020 FF26	25 - 31	0.29	0.0537	Inf	5.397	<.0001
	31 - 32	0.114	0.07	Inf	1.63	0.1032
	32 - 33	0.607	0.1031	Inf	5.886	<.0001
	33 - 34	0.573	0.1559	Inf	3.674	0.0002
	34 - 35	1.04	0.2629	Inf	3.958	0.0001
wp/tsl FF21	25 - 31	0.141	0.0351	Inf	4.016	0.0001
	31 - 32	1.014	0.0973	Inf	10.421	<.0001
	32 - 33	1.175	0.2099	Inf	5.598	<.0001
	33 - 34	2.185	0.615	Inf	3.553	0.0004
	34 - 35	0	0.8279	Inf	0	1
wp/tsl FF26	25 - 31	0.14241	0.0296	Inf	4.814	<.0001
	31 - 32	-0.00888	0.0342	Inf	-0.26	0.795
	32 - 33	1.30568	0.0862	Inf	15.155	<.0001
	33 - 34	2.12389	0.2777	Inf	7.649	<.0001
	34 - 35	0.69315	0.461	Inf	1.504	0.1327
wp/tsl (EgII) FF21	25 - 31	0.1867	0.0548	Inf	3.407	0.0007
	31 - 32	-0.0346	0.0656	Inf	-0.527	0.5982
	32 - 33	2.0488	0.2577	Inf	7.95	<.0001
	33 - 34	2.3136	0.8842	Inf	2.617	0.0089
	34 - 35	0	1.1978	Inf	0	1
wp/tsl (EgII) FF26	25 - 31	0.1059	0.0228	Inf	4.643	<.0001
	31 - 32	-0.0178	0.0259	Inf	-0.687	0.4923
	32 - 33	1.0905	0.0643	Inf	16.948	<.0001
	33 - 34	3.2108	0.3649	Inf	8.799	<.0001
	34 - 35	-0.3365	0.4705	Inf	-0.715	0.4745
D53-3-28 FF21	25 - 31	0.08091	0.0305	Inf	2.651	0.008
	31 - 32	-0.00251	0.0372	Inf	-0.068	0.9461
	32 - 33	0.10717	0.0452	Inf	2.371	0.0177
	33 - 34	0.5454	0.0868	Inf	6.281	<.0001
	34 - 35	0.95937	0.1756	Inf	5.464	<.0001
D53-3-28 FF26	25 - 31	0.04856	0.0337	Inf	1.44	0.1498
	31 - 32	-0.00846	0.0387	Inf	-0.218	0.8272
	32 - 33	0.11328	0.0503	Inf	2.25	0.0245
	33 - 34	0.41903	0.0952	Inf	4.404	<.0001
	34 - 35	0.72226	0.1766	Inf	4.089	<.0001
D53-1 FF26	25 - 31	0.1565	0.0267	Inf	5.858	<.0001
	31 - 32	-0.0362	0.0325	Inf	-1.115	0.2648
	32 - 33	1.595	0.113	Inf	14.11	<.0001
	33 - 34	2.8397	0.5136	Inf	5.529	<.0001
	34 - 35	0	0.7092	Inf	0	1

* Inf = Infinite degrees of freedom. Z test used for pairwise comparison is not affected by the number of observations.
